# Neonatal Bacteremia Caused by Herbaspirillum huttiense in a Neonatal Intensive Care Unit: A Case Series

**DOI:** 10.7759/cureus.109597

**Published:** 2026-05-25

**Authors:** Asma Amarai, Almahdi Afroukh, Taoufik Ben Houmich, Asmae Lamrani Hanchi, Nabila Soraa

**Affiliations:** 1 Department of Microbiology, Faculty of Medicine and Pharmacy, Cadi Ayyad University, Mohammed VI University Hospital, Marrakesh, MAR

**Keywords:** case series, herbaspirillum huttiense, neonatal bacteremia, neonatal intensive care unit, opportunistic pathogen, rare infection

## Abstract

Background: *Herbaspirillum huttiense* is a rare environmental Gram-negative bacillus increasingly recognized as an emerging opportunistic pathogen in humans. Due to its phenotypic similarities with other non-fermenting Gram-negative bacteria, it is frequently misidentified, and data on its clinical characteristics and management remain limited.

Methods: This was a retrospective case series study conducted over one month (March 2023) in the Microbiology Department of Mohammed VI University Hospital, Marrakesh, Morocco. The study included neonates hospitalized in the Neonatal Intensive Care Unit (NICU) with at least one positive blood culture for *Herbaspirillum* spp. Patients with negative blood cultures or with bloodstream infections caused by pathogens other than *Herbaspirillum* spp. were excluded. Clinical and biological characteristics, including signs of sepsis, were collected retrospectively from medical records for descriptive analysis. Identification was performed using matrix-assisted laser desorption/ionization time-of-flight mass spectrometry (MALDI-TOF MS, BD, Franklin Lakes, NJ). Antimicrobial susceptibility testing was performed using the automated Phoenix M50 system (BD Diagnostics, Sparks, MD) in accordance with EUCAST guidelines. Only descriptive statistics were used.

Results: Six neonates were included in the study, with an equal sex distribution (three males and three females). The majority were preterm infants (83.3%), with a mean gestational age of 34.2 weeks (range: 30.4-37.4 weeks) and a mean birth weight of 2.3 kg (range: 1.48-3.20 kg). Most deliveries were vaginal (83.3%). The main reasons for admission were neonatal respiratory distress (66.7%), followed by neonatal seizures and hypoglycemia (16.7% each). Comorbidities included severe perinatal asphyxia, persistent pulmonary hypertension of the newborn, and omphalocele. During hospitalization, the neonates developed clinical signs of sepsis after a mean delay of seven days (range: days four and nine), presenting with fever, tachycardia, and skin mottling. Biologically, patients developed leukopenia (mean leukocyte count: 9,500/mm³), elevated C-reactive protein (CRP) levels (mean: 89 mg/L), and thrombocytopenia (mean platelet count: 77,833/mm³). Blood cultures became positive after incubation, yielding Gram-negative, oxidase-positive bacilli identified as *H. huttiense*. The isolates were susceptible to carbapenems, cefepime, and amikacin, but resistant to colistin. All patients had a favorable outcome, with a 100% survival rate and a mean hospital stay of 16 days.

Conclusion: *H. huttiense* is an emerging opportunistic pathogen in neonatal infections, often associated with diagnostic challenges due to misidentification. Advanced diagnostic tools such as MALDI-TOF significantly improve detection. Although no standardized treatment guidelines exist, most isolates appear susceptible to broad-spectrum antibiotics, with favorable clinical outcomes under appropriate therapy.

## Introduction

*Herbaspirillum *spp. are non-fermenting, aerobic, Gram-negative bacilli with a helical or curved morphology, belonging to the class Betaproteobacteria and the order Burkholderiales [1,2].

Traditionally, these microorganisms are considered environmental bacteria, widely present in soil and aquatic environments, particularly in the plant rhizosphere [3]. However, advances in microbiological identification techniques have gradually revealed their potential involvement in human infections, thereby increasing their clinical importance in microbiology [2].

To date, only a few studies and case reports have described infections caused by *Herbaspirillum huttiense*, particularly in pediatric populations [4]. Available data remain limited regarding its epidemiology, clinical spectrum, antibiotic susceptibility profiles, and therapeutic management [1,5]. Reported cases show a wide variety of clinical manifestations, ranging from respiratory infections to severe bacteremia, reflecting the opportunistic behavior of this microorganism [4,6]. In addition, several cases were initially misdiagnosed or misidentified as other non-fermenting Gram-negative bacilli, highlighting the diagnostic difficulties of this rare pathogen [7].

This study aims to describe a case series of neonatal bacteremia caused by *H. huttiense *in six newborns admitted to a Neonatal Intensive Care Unit (NICU), focusing on clinical characteristics, biological findings, antimicrobial susceptibility patterns, and the diagnostic contribution of matrix-assisted laser desorption/ionization time-of-flight mass spectrometry (MALDI-TOF MS) in identifying this rare emerging pathogen.

## Materials and methods

Study design and population

This was a retrospective case series study conducted over one month (March 2023) in the Microbiology Department of the Mohammed VI University Hospital in Marrakesh, Morocco. The study included neonates hospitalized in the NICU who developed bacteremia due to *Herbaspirillum *spp. during their hospital stay. A total of six neonates were included.

Inclusion criteria

Neonates hospitalized during the study period (March 2023) with at least one positive blood culture for *Herbaspirillum *spp. were included.

Exclusion criteria

Neonates with negative blood cultures or with positive blood cultures for pathogens other than *Herbaspirillum *spp. were excluded.

Data collection

Clinical data were collected retrospectively from medical records, including gestational age, birth weight, sex, reason for admission, and clinical signs of sepsis, including temperature instability (fever or hypothermia), poor feeding, lethargy, irritability, respiratory distress (tachypnea, apnea, grunting), and cardiovascular instability (tachycardia, poor perfusion, hypotension). Laboratory parameters included C-reactive protein, leukocyte count, and platelet count, along with respiratory support, antimicrobial therapy, microbiological results, and clinical outcome.

Microbiological methods

Bacterial identification was performed using MALDI-TOF MS (BD, Franklin Lakes, NJ), used as the primary identification method without systematic conventional comparative identification. Antimicrobial susceptibility testing was performed using the automated Phoenix M50 system (BD Diagnostics, Sparks, MD) in accordance with the European Committee on Antimicrobial Susceptibility Testing (EUCAST) guidelines [8].

Statistical analysis

Data were analyzed using Microsoft Excel 2021 (Microsoft® Corp., Redmond, WA). Only descriptive statistics were performed. Quantitative variables were expressed as means, and categorical variables as percentages. No inferential statistical analyses were conducted.

All neonates who met the inclusion criteria during the study period were included, representing an exhaustive case series (n = 6). No sample size calculation or sampling method was applied. Given the limited number of cases, no analysis of confounding factors was performed.

Ethical approval

The requirement for ethical approval was waived by the institutional review board. Only anonymized data were used, and the study focused on microbiological findings related to the pathogen, without any direct patient involvement.

## Results

Six newborns were included during the study period, with an equal sex distribution (three males, three females). Five patients (83.3%) were preterm, with a mean gestational age of 34.2 weeks (range: 30.4-37.4). The mean birth weight was 2.3 kg (range: 1.48-3.20 kg). Most deliveries were vaginal (83.3%; n = 5), with one cesarean section (16.7%; n = 1). The primary reasons for admission were neonatal respiratory distress (n = 4; 66.7%), followed by neonatal seizures (n = 1; 16.7%) and hypoglycemia (n = 1; 16.7%) (Table 1).

**Table 1:** Characteristics of neonates with Herbaspirillum spp. bacteremia.

Patient	Gestational age (GA) at birth (weeks)	Birth weight (kg)	Mode of delivery	Sex	Admission diagnosis	Respiratory support
1	35 weeks	2.5	Normal vaginal delivery	M	Neonatal respiratory distress associated with persistent pulmonary hypertension of the newborn	oxygen via nasal cannula
2	34 weeks and 2 days	2.26	Normal vaginal delivery	M	Neonatal seizures secondary to perinatal asphyxia (Sarnat stage III)	oxygen via nasal cannula
3	30 weeks and 4 days	1.48	Normal vaginal delivery	F	Neonatal respiratory distress	Continuous positive airway pressure (CPAP) → intubation with sedation
4	34 weeks and 2 days	2.38	Normal vaginal delivery	F	Neonatal respiratory distress	Continuous positive airway pressure (CPAP)
5	37 weeks and 4 days	3.20	Delivery by cesarean section	M	Hypoglycemia associated with an omphalocele	Oxygen via nasal cannula
6	34 weeks	1.95	Normal vaginal delivery	F	Neonatal respiratory distress	Continuous positive airway pressure (CPAP)

Comorbidities were observed in some neonates. The patient admitted for seizures (Patient 2) had severe perinatal asphyxia (Sarnat stage III). Transfontanellar ultrasound revealed a cystic lesion located beneath the floor of the frontal horn of the right lateral ventricle, associated with bilateral periventricular white matter hyperechogenicity, suggestive of hypoxic-ischemic injury. Among the neonates admitted for neonatal respiratory distress, Patient 1 presented with persistent pulmonary hypertension of the newborn; transthoracic echocardiography showed dilatation of the pulmonary artery, a small patent foramen ovale with right-to-left shunt, and right ventricular hypertrophy. Furthermore, in the neonate presenting with hypoglycemia (Patient 5), an omphalocele was diagnosed; abdominal ultrasound showed herniation of part of the left lobe of the liver, the gallbladder, and intestinal loops, associated with a moderate peritoneal effusion.

At admission, CRP levels ranged from 3.83 to 25 mg/L, with a mean of 11.3 mg/L. Leukocyte counts ranged from 8,520 to 17,850/mm³, with a mean of 10,765/mm³, without marked leukocytosis. Platelet counts ranged from 80,000 to 212,000/mm³, with a mean of 168,000/mm³.

Non-invasive ventilation using continuous positive airway pressure (CPAP) was used in three patients, while three others received low-flow oxygen therapy via nasal cannula. One patient required invasive mechanical ventilation with endotracheal intubation. None of the patients had a central venous catheter or umbilical catheter during hospitalization.

During hospitalization, the neonates developed clinical signs of sepsis after a mean delay of seven days (range: days 4-9). Clinical manifestations included tachycardia, skin mottling, and febrile peaks. Jaundice was observed in one patient (16.7%). On biological assessment, all patients presented with leukopenia, with a mean leukocyte count of 9,500/mm³ (range: 4,570-13,030/mm³); elevated C-reactive protein (CRP) levels, with a mean of 89 mg/L (range: 41.9-147 mg/L); and thrombocytopenia, with platelet counts ranging from 14,000 to 118,000/mm³, corresponding to a mean of 77,833/mm³.

**Figure 1:**
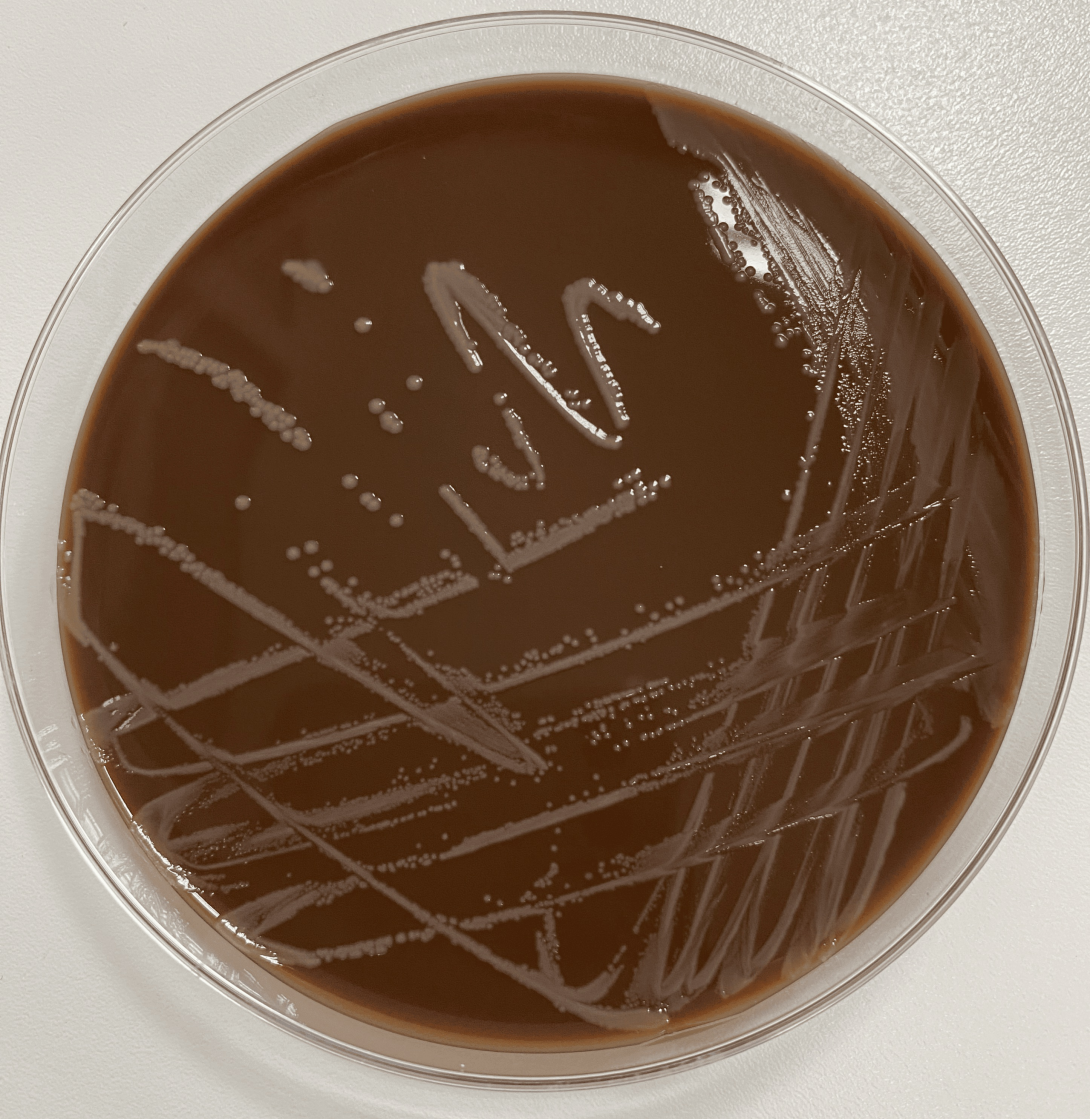
Culture of Herbaspirillum huttiense on chocolate agar after 48 hours of incubation.

Blood cultures were collected from neonates using pediatric blood culture bottles and incubated at 37°C in an automated blood culture system (BACTEC FX system; Becton Dickinson, Sparks, MD). All positive blood cultures yielded Gram-negative, oxidase-positive bacilli (Figure 1). The isolates were directly identified as *H. huttiense* using MALDI-TOF MS.

Antibiotic susceptibility testing was performed using the automated system PHOENIX M50. The isolates were susceptible to imipenem, meropenem, cefepime, amikacin, and the trimethoprim/sulfamethoxazole combination. All strains were resistant to colistin (Table 2).

**Table 2:** Minimum inhibitory concentration (MIC) results of Herbaspirillum huttiense.

Antibiotics	MIC (mg/L)
Ampicillin	≤ 4
Piperacillin-tazobactam	≤ 4/4
Cefepime	≤ 1
Imipenem	≤ 0.25
Meropenem	≤ 0.25
Levofloxacin	1
Amikacin	≤ 8
Gentamicin	4
Cotrimoxazole	≤ 2/38
Colistin	>4

Initial empirical antibiotic therapy consisted of third-generation cephalosporins (C3G) combined with gentamicin. Antibiotic therapy was subsequently adapted following microbiological results, with a switch to meropenem and amikacin. Adjunctive treatments were required, including platelet transfusion for thrombocytopenia and phototherapy for neonatal jaundice. Phenobarbital was administered to the patient with seizures, with a loading dose of 20 mg/kg intravenously, followed by a maintenance dose of 5 mg/kg/day.

The clinical outcome was favorable in all patients, with a survival rate of 100%. Clinical evolution was characterized by improvement in the general condition, hemodynamic stabilization, and regression of infectious signs under treatment, with progressive normalization of biological parameters. The mean duration of hospitalization was 16 days.

## Discussion

*Herbaspirillum *spp. are non-fermenting, Gram-negative, strictly aerobic bacilli characterized by a curved or spiral morphology, motility via polar flagella, and positive oxidase, catalase, and urease activities [2]. The genus *Herbaspirillum *was initially described by Baldani et al. in 1996 [2].

From an ecological perspective, *Herbaspirillum *species are predominantly environmental bacteria commonly associated with plants, colonizing their root systems; they have also been isolated from aquatic environments, including wells and groundwater sources [3]. These microorganisms are known to enhance plant growth by producing phytohormones and play a role in biological nitrogen fixation, contributing to soil fertility and plant development [3,5].

Infections caused by *H. huttiense *are rarely reported in the literature; most cases have been described as isolated cases in different areas of the world, underscoring the sporadic nature of this pathogen [4]. Clinical manifestations are heterogeneous, ranging from respiratory infections associated with bacteremia to severe cases of sepsis and septic shock, even in immunocompetent patients [4,6]. This rarity suggests that *H. huttiense *is an emerging opportunistic pathogen whose true incidence is likely underestimated [1,4,9]. In addition, neonatal conditions, such as prematurity and low birth weight, have been suggested as potential predisposing factors [10].

In neonates, sepsis presents with non-specific clinical signs, including temperature instability (fever or hypothermia), feeding difficulties, lethargy, irritability, and respiratory distress (tachypnea, apnea, grunting) [11]. It may also be associated with cardiovascular instability (tachycardia, poor perfusion, hypotension) and, less frequently, neurological or gastrointestinal signs [11].

Misidentification remains a major diagnostic challenge for *H. huttiense*, as phenotypic similarities with the *Burkholderia cepacia *complex often lead to misidentification when conventional biochemical methods are used [7]. The introduction of MALDI-TOF MS has significantly improved the speed and accuracy of identifying these rare bacteria compared to conventional biochemical methods [5,6]. In addition, molecular techniques, such as 16S rRNA gene sequencing and next-generation sequencing (NGS), can be used as complementary methods to enhance the accuracy of bacterial identification [4].

In this study, all cases were identified in the same NICU during March 2023; however, the available data do not allow confirmation of transmission between them or identification of a common source.

*H. huttiense *is generally described as being susceptible to several antibiotics, including β-lactams (e.g., piperacillin/tazobactam), carbapenems, such as meropenem, trimethoprim-sulfamethoxazole, and amikacin [4,6,12]. Antibiotic susceptibility profiles can also help differentiate *H. huttiense *from the *B. cepacia *complex, which is generally associated with multidrug resistance [7].

Regarding antimicrobial therapy, available data suggest that piperacillin/tazobactam and meropenem are associated with favorable clinical outcomes, which is in line with our findings, as all patients responded favorably to meropenem-based therapy [4].

Study strengths

The main strength of this study lies in the description of a rare and emerging neonatal pathogen, *H. huttiense*,in a vulnerable population of neonates admitted to an NICU. This observation contributes valuable clinical and microbiological data to the still-limited literature.

Another important strength is the reliable microbiological identification using MALDI-TOF MS, highlighting the value of advanced diagnostic techniques for the accurate identification of unusual Gram-negative pathogens, which may otherwise be difficult to identify using routine laboratory methods.

In addition, the study provides a detailed description of the clinical presentation, biological findings, and antimicrobial susceptibility profiles, which may help guide the management of similar cases.

Study limitations

This study has limitations inherent to its design. Its retrospective and single-center nature, together with the small sample size (n = 6), limits the statistical power and generalizability of the findings. In addition, some clinical and microbiological data were not systematically available in the medical records, which restricted more detailed analysis. Although the cases were identified during the same period (March 2023), no detailed spatial or epidemiological analysis was performed. The absence of systematic environmental investigation and molecular confirmation (e.g., 16S rRNA sequencing) does not allow definitive identification of the source or transmission pathways, nor confirmation of a transmission link between the cases. Finally, only descriptive statistics were used due to the limited sample size, and no inferential statistical analysis was performed.

## Conclusions

*H. huttiense *is an uncommon opportunistic Gram-negative bacillus increasingly recognized due to advances in microbiological identification techniques. In this retrospective case series, we describe six cases of *H. huttiense *bacteraemia in an NICU, illustrating its occurrence in a highly vulnerable neonatal population. Accurate identification using MALDI-TOF MS was essential for diagnosis, highlighting the challenges associated with identifying rare non-fermenting Gram-negative bacilli using conventional microbiological approaches, as reported in the literature.

The clinical outcomes observed in our series, together with antimicrobial susceptibility data, suggest that targeted antibiotic therapy was associated with favorable outcomes. However, due to the retrospective design, the small sample size, and the absence of molecular and environmental investigations, these findings should be interpreted as descriptive observations and do not allow definitive conclusions regarding transmission dynamics or broader epidemiological implications.

## References

[1] Özen S, Kanik Yüksek S, Dinç B (2024). Catheter-related infections in pediatric patients due to a rare pathogen: Herbaspirillum huttiense. Pediatr Infect Dis J.

[2] Baldani JI, Pot B, Kirchhof G (1996). Emended description of Herbaspirillum; inclusion of (Pseudomonas) rubrisubalbicans, a milk plant pathogen, as Herbaspirillum rubrisubalbicans comb. nov.; and classification of a group of clinical isolates (EF group 1) as Herbaspirillum species 3. Int J Syst Bacteriol.

[3] Rothballer M, Eckert B, Schmid M (2008). Endophytic root colonization of gramineous plants by Herbaspirillum frisingense. FEMS Microbiol Ecol.

[4] Ruiz de Villa A, Alok A, Oyetoran AE, Fabara SP (2023). Septic shock and bacteremia secondary to Herbaspirillum huttiense: a case report and review of literature. Cureus.

[5] Li X, Bao X, Qiao G (2022). First study of bacteremia caused by Herbaspirillum huttiense in China: a brief research report and literature review. Front Cell Infect Microbiol.

[6] Liu C, Kwon MJ, Kim M, Byun JH, Yong D, Lee K (2019). Septicemia caused by Herbaspirillum huttiense secondary to pneumonia. Ann Lab Med.

[7] Wang Q, Cai X, Zhang L (2024). Uncommon pathogen misidentification of Herbaspirillum huttiense as Burkholderia cepacia in bacteremia: a case report. Lab Med.

[8] Amara M, Barraud O, Cadenet J (2025). Antibiotic Susceptibility Testing Committee of the French Society for Microbiology (CA-SFM): Recommendations 2025, V.1.1 July [Article in French]. Recommendations 2025. Société Française de Microbiologie, Paris.

[9] Chemaly RF, Dantes R, Shah DP (2015). Cluster and sporadic cases of Herbaspirillum species infections in patients with cancer. Clin Infect Dis.

[10] Tas M, Turkyilmaz C, Bozdayi G, Tezer H, Koc E (2025). Case report: an unexpected Herbaspirillum huttiense bacteremia in the neonatal intensive care unit. Z Geburtshilfe Neonatol.

[11] Kariniotaki C, Thomou C, Gkentzi D, Panteris E, Dimitriou G, Hatzidaki E (2025). Neonatal sepsis: a comprehensive review. Antibiotics (Basel).

[12] Abreu-Di Berardino M, Rodríguez-Czaplicki E, Sánchez-Hellín V (2019). Herbaspirillum huttiense pneumonia in a patient with essential thrombocythaemia. Rev Esp Quimioter.

